# Impact of Clinical Pharmacy Services on KAP and QOL in Cancer Patients: A Single-Center Experience

**DOI:** 10.1155/2015/502431

**Published:** 2015-11-30

**Authors:** Yan Wang, Huimin Wu, Feng Xu

**Affiliations:** ^1^Department of Pharmacy, Fengxian Hospital, Southern Medical University, Shanghai 201400, China; ^2^Department of Pharmacy, Guangdong Medical University Affiliated Hospital, Zhanjiang 524001, China

## Abstract

This study was to evaluate the efficacy of pharmaceutical intervention (PI) on chemotherapy knowledge-attitude-practice (KAP) and quality of life (QOL) in cancer patients. A prospective, randomized, controlled study was carried out at Oncology Ward in a tertiary hospital affiliated to Southern Medical University, China. Eligible patient was randomly assigned to pharmaceutical intervention (PI) group or control group. Each patient in PI group was given information booklets and was given 30 min face-to-face medication education and psychological counseling by clinical pharmacists, 2 sessions per week for 2 months. Patients in control group only received conventional treatment. All participants were asked to complete a structured Chemotherapy KAP Questionnaire and QOL Questionnaire at pre- and poststudy time. A total of 149 cancer patients (77 in PI group and 72 in control group) completed the study. The baseline scores of KAP and QOL in 2 groups were similar. At the end of study, only knowledge score was significantly increased; meanwhile no difference existed for attitude, practice, and QOL scores in control group; both KAP scores and QOL score were significantly increased in PI group. As for the between-group comparison, both KAP scores and QOL score in PI group were significantly higher than those in control group. In conclusion, pharmaceutical intervention has a positive role in increasing chemotherapy-related knowledge, improving patients' positive emotions, dealing with chemotherapy adverse reactions, and improving the quality of life of patients.

## 1. Introduction

Primary malignant cancer is currently the leading cause of cancer-related mortality worldwide. About 14 million individuals are newly diagnosed with cancer annually in the world, and more than 8 million died of cancer [1]. During the past decades, great progress has been made for the diagnosis and treatment of cancer. Chemotherapy is one of the treatment methods for malignant cancer. However, most anticancer drugs have varying degrees of toxicity and adverse drug reactions (ADRs) often occur during the chemotherapy. ADRs usually reduce the quality of life of cancer patients, prolong their stay time, and increase patients' financial burden; meanwhile severe ADRs often interrupt the chemotherapy and even lead to failure. It is worth noting that fear for chemotherapy toxicity and expectations for chemotherapy outcome bring patients into a “trend-avoid” psychological conflict [2].

Evidences showed that, in cancer care, clinical pharmacists contribute to improving prevention and management of drug-related problems [3]. The pharmaceutical care of clinical pharmacists helps patients to understand medication [4]. A cross-sectional survey displayed that patients are willing to receive counseling services of pharmacist regularly during chemotherapy treatment and they may like to pay for the services [5]. At present, we have full-time oncology clinical pharmacists in China, who are mainly engaged in therapeutic drug monitoring, patient medication education, psychological counseling, and other services [6–9]. Although there are many studies evaluating the patient benefits from pharmaceutical intervention (PI) in Oncology Ward, there is no validated published survey to evaluate the efficacy of PI on chemotherapy knowledge-attitude-practice (KAP).

## 2. Patients and Methods

### 2.1. Eligible Patients

Patients were recruited from Oncology Ward in a tertiary hospital of China. The eligible patients (1) were aged between 18 and 70 years; (2) were diagnosed with cancer by cytology, imaging, and pathology tests; (3) accepted chemotherapy less than 3 cycles; (4) had a performance status score of less than 3; (5) had a life expectancy of more than 6 months; and (6) were able to communicate verbally. Patients who had a major psychiatric disease or any other very serious illness were excluded. Patient general condition was assessed by ZUBROD-ECOG-WHO Performance Status (ZPS) scores ranging from 0 to 5 [10].

### 2.2. Ethics Statement

The study was approved by Hospital Ethics Committees, and all eligible patients agreed to participate in the study and signed an informed consent.

### 2.3. Study Design and Process

A prospective, randomized, controlled study was designed; eligible patients were randomly assigned to pharmaceutical intervention (PI) group and control group by random number table. Patients in PI group received comprehensive pharmaceutical care and our self-compiled booklet—*Cancer Patients Medication Knowledge Guide*. The booklet provides patients with the following medical information: (1) chemotherapy purpose; (2) chemotherapy preparation and assessment; (3) prevention and management of adverse drug reactions; and (4) cautions of oral chemotherapy drugs. The comprehensive pharmaceutical care includes face-to-face medication education and psychological counseling service provided by clinical pharmacist, 2 sessions per week for 2 months. Each session lasted for approximately 30 minutes. Medication education focuses on rational drug use, reasonable dietary guidance, and others. Clinical pharmacists kept full contact with patients—mobile phone, email, and wechat—during all the period. Meanwhile, patients were encouraged to exchange medication information to other healthcare professionals as well as the family members and friends. Patients in control group only received routine medical service.

### 2.4. Data Collection and Study Instruments

The demographics and clinical characteristics data were collected. The following study instruments—European Organization for Research and Treatment of Cancer Core Quality of Life Questionnaire (EORTC QLQ-C30) and Questionnaire on Chemotherapy Knowledge-Attitude-Practice (KAP)—were used. EORTC QLQ-C30 consists of 30 questions which make up 5 functional scales (physical, role, emotional, cognitive, and social), 3 symptom scales (fatigue, nausea/vomiting, and pain), 6 individual items (dyspnea, insomnia, appetite loss, constipation, diarrhea, and financial difficulties), and an overall QOL scale. The raw scores were linearly transformed into scores ranging from 0 to 100 [11–13]. A structured Chemotherapy KAP Questionnaire was developed on the basis of [14, 15], which consisted of 3 dimensions (knowledge, attitude, and practice) (the Chemotherapy Knowledge-Attitude-Practice Questionnaire is shown below). A higher score represents rich knowledge, positive attitude, and behavior.

*Knowledge Dimension*

1. Do you know the whole chemotherapy regimen before the chemotherapy?
  (A) Yes
  (B) No
2. Do you know the name of the chemotherapy drugs to you?
  (A) Yes
  (B) No
3. Do you think it was harmful to the surrounding tissues while the chemotherapy drugs leak out of the blood vessels?
  (A) Yes
  (B) No
  (C) I don't know
4. What should you do if you have nausea and vomiting during the chemotherapy?
  (A) Eat frequent but smaller meals
  (B) Avoid nasty smell, such as smoke flavor or perfume
  (C) Eat greasy food
  (D) Take a deep breath slowly, while you felt like to vomit
5. Do you think it would be regenerated that the hair loss caused by chemotherapy?
  (A) Yes
  (B) No
  (C) I don't know
6. What should you do if your temperature was over 38°C during the chemotherapy?
  (A) Take antipyretic analgesics, such as aspirin
  (B) Tell your doctors or nurse
  (C) Don't take measures
  (D) I don't know
7. What should you do if your white blood cell reduced during the chemotherapy?
  (A) Don't contact with cold, flu, measles or the chicken pox patient
  (B) Get vaccinated for influenza and pneumonia
  (C) Play in public places
  (D) I don't know
8. In following, which were the phenomenon of low platelets? (Can select more)
  (A) Unexplained cyanosis of skin
  (B) Gums and nose bleeding
  (C) Nausea
  (D) I don't know
9. What should you do if your platelets reduced during the chemotherapy? (Can select more)
  (A) Brush your teeth with a soft bristle toothbrush
  (B) Play basketball
  (C) Avoid injuries such as damage, burns
  (D) I don't know
10. What should you do if you had oral cavity ulcer during the chemotherapy?
  (A) Eat hot food
  (B) Eat warm food
  (C) Eat spicy food
  (D) I don't know
11. What should you do if there had a peculiar taste in your mouth during the chemotherapy? (Can select more)
  (A) Eat frequent but smaller meals
  (B) Brush teeth or rinses mouth before meals
  (C) Eat with metal tableware
  (D) I don't know
12. What should you do if you had a diarrhea during the chemotherapy?
  (A) Drink milk
  (B) Eat high-fiber food such as fresh fruit and vegetable
  (C) Eat low-fiber food such as boiled egg, fish
  (D) I don't know
13. What should you do if you had constipation during the chemotherapy?
  (A) Drink plenty of water
  (B) Take laxative
  (C) Lay on bed to rest
  (D) I don't know
14. Would you need to drink more water during the chemotherapy?
  (A) Yes
  (B) No
  (C) I don't know.

*Attitude Dimension*

(15) Most of the chemotherapy-induced side effects will disappear after the chemotherapy, what is your opinion on this view?
  (A) Completely agree
  (B) Agree
  (C) Neutral
  (D) Disagree
  (E) Strongly disagree
(16) The chemotherapy-induced nausea and vomiting could be prevented or reduced, what is your opinion on this view?
  (A) Completely agree
  (B) Agree
  (C) Neutral
  (D) Disagree
  (E) Strongly disagree
(17) The chemotherapy-induce adverse reactions could be reduced though self-care, what is your opinion on this view?
  (A) Completely agree
  (B) Agree
  (C) Neutral
  (D) Disagree
  (E) Strongly disagree
(18) It would cause “addiction” that to take analgesics for pain, what is your opinion on this view?
  (A) Completely agree
  (B) Agree
  (C) Neutral
  (D) Disagree
  (E) Strongly disagree
(19) Would you want to receive health education on chemotherapy?
  (A) Completely agree
  (B) Agree
  (C) Neutral
  (D) Disagree
  (E) Strongly disagree.

*Practice Dimension*

(20) Do you drink (Drinking alcoholic beverages at least once a week, and lasting more than a year)?
  (A) Drinking
  (B) Drunk in the past, but not now
  (C) Never
(21) Do you smoke, (Smoking more than 1 cigarette daily, and lasting for more than 1 year)?
  (A) Smoking
  (B) Smoked in the past, but not now
  (C) Never smoke
(22) What should you do if the chemotherapy drugs leak out of the blood vessels?
  (A) Call the nurse immediately
  (B) Slow infusion rate to continue to infuse
  (C) No matter, do not pay attention
  (D) I don't know
(23) Which of the following couldn't be appropriate if the chemotherapy caused hair loss?
  (A) Use a mild shampoo
  (B) Wear a hat to prevent sunburn.
  (C) Perm the hair to be fluffy
  (D) I don't know
(24) What should you do if you felt fatigue during the chemotherapy?
  (A) Lie on the bed to rest continuously
  (B) Save energy, combine work with adequate rest
  (C) Drinking
  (D) I don't know
(25) Would you return to the hospital on time for the chemotherapy on medical advice?
  (A) Yes
  (B) No
  (C) I don't know.

### 2.5. Statistics

All of the data were analyzed by the statistical software SPSS (version 13.0). Chi-square test was used to analyze classification data of two groups. The independent samples *t*-test was used to compare the means of two groups before intervention. A paired *t*-test was used to compare the changes before and after intervention within each group. ANCOVA (Analysis of Covariance) was used to compare the changes between two groups after intervention. The test level was as follows: *α* = 0.05, with 95% confidence interval.

## 3. Results

A total of 155 cancer patients were recruited and randomized into control group (76) and PI group (79). During the study period, 4 patients in control group and 2 patients in PI group dropped out due to death or advancing illness. Finally 149 patients (72 in control group and 77 in PI group) completed the study. There were 84 males and 65 females, with mean age of 49 years (range from 18 to 70). There were no differences for demographic and clinical characteristics between 2 groups (*P* > 0.05, Table 1).

In this study, we designed Chemotherapy KAP Questionnaire which consists of 3 dimensions with 25 items. There are 14, 5, and 6 items for knowledge, attitude, and practice, respectively. A higher score represents rich knowledge and positive attitude and behavior. In the knowledge dimension, a correct answer gets 1 point and the others get 0 points for the single-choice question; the correct answers get 1 point and the others get 0 points for the multiple-choice question. The total scores are ranging from 0 to 19. In the attitude dimension, each item is composed of 5 levels' answers (completely agree, agree, neutral, disagree, and strongly disagree) corresponding to 1, 2, 3, 4, and 5 score. The total scores are ranging from 5 to 25. In practice dimension, for 20th and 21st question choosing either (B) or (C) gets 1 point; for the other questions the correct answer gets 1 point. The total scores are ranging from 0 to 6. In the pilot study, Cronbach's alpha was 0.71, and Pearson correlation coefficient was 0.93, so the internal consistency of the questionnaire was good.

The baseline scores of KAP and overall QOL in 2 groups were similar. At the end of study, only knowledge score was significantly increased; meanwhile no difference existed for attitude, practice, and overall QOL scores in control group; both KAP scores and overall QOL score were significantly increased in PI group. As for the between-group comparison, both KAP scores and overall QOL score in PI group were significantly higher than those in control group (*P* < 0.05, Tables 2 and 3).

In the aspects of QOL data specifically 5 functional scales were increased after the study; however, the increases of emotional and cognitive function were significantly in the PI group (*P* < 0.05, Table 3). Three symptom scales and 6 individual measurement project scores were decreased at the end of study; however, only fatigue, nausea/vomiting, pain, sleep disturbance, constipation, and diarrhea were significantly decreased in the PI group (*P* < 0.05, Table 3). Meanwhile there were no significant differences for all pre- and poststudy scale scores in control group.

## 4. Discussion

Although there are many studies evaluating the patient benefits from pharmaceutical care service in Oncology Ward, there is no report to evaluate the efficacy of pharmaceutical care on chemotherapy knowledge-attitude-practice (KAP). The purposes of pharmaceutical care service in oncology include the following: optimizing chemotherapy therapy regimes, minimizing side effect/toxicity, providing medication education and psychological support, and improving patients' outcome and quality of life. The results showed that our pharmaceutical interventions have obtained obvious efficacy in cancer patients—effectively enhancing chemotherapy-related knowledge, improving positive emotions, and correcting some bad behaviors. It is interesting to note that chemotherapy-related knowledge was also significantly increased in control group. We speculate that although these patients did not receive comprehensive medication education, they could still obtain some beneficial information from other resources—healthcare professionals, family member, friends, and currently the Internet.

This study confirmed that pharmaceutical care service could improve the overall QOL which is consistent with [16–27]. Naeem et al. found that psychological health intervention could change inappropriate cognitive style and improve positive emotions and behavior disorders [16]. However, currently very few psychologists work in Oncology Ward in China's hospitals. The pharmacist, as a member of the multidisciplinary team, can contribute significantly to reduce medication-related problems and to optimize chemotherapy for cancer patients. Based on our results, clinical pharmacist service could also reach the similar outcome. In the present study, physical function, role function, and social function were not significantly improved in PI group. It is probably due to the fact that the intervention time was too short.

Yun et al. demonstrated that a 12-week Internet-based education program was effective for disease-free cancer survivors with cancer-related fatigue [17]. Do et al. showed that a 4-week multimodal rehabilitation program improved the physical symptoms and QOL and reduced fatigue in patients with breast cancer [18]. Many studies showed that education, counseling, behavioral interventions, relaxation interventions, and nutritional support could significantly allay or alleviate chemotherapy-led nausea and vomiting and pain [19, 20]. Our results are consistent with the above-mentioned literatures. The patients reduced fatigue, the symptoms of nausea and vomiting, and pain as they followed clinical pharmacists' advice.

Sleep disturbance is one of the most frequent symptoms complained about by cancer patients. About 15% to 90% of cancer patients and survivors claimed sleep disorders such as excessive daytime napping, difficulty in falling asleep, frequent interruptions of sleep, and waking up too early [21–23]. Sleep disturbance not only caused depression patients, interfered with their daily activities, and reduced quality of life but also impaired cancer-treatment adherence, affected the treatment outcomes, and even possibly increased patients' mortality [24, 25]. Constipation and diarrhea are highly prevalent in cancer patients which are unpleasant physical symptoms and negatively influence patients' quality of life. There are many possible causes of cancer-related constipation and diarrhea, including physiological factors, psychological factors, drug, diet, and cancer treatment [26, 27]. In a word, quality of life for cancer patients can be significantly improved through comprehensive care [28, 29]. Our data also proved that medication education and nonpharmacological intervention (e.g., cognitive behavioral interventions, relaxation interventions, and music therapy) by clinical pharmacists could effectively improve the quality of sleep and reduced cancer-related constipation and diarrhea of cancer patients.

It is worth mentioning that clinical pharmacists were well accepted as patient medication educator and psychological consultant. In PI group, most consulting problems from patients were about side effects of chemotherapy. Cancer patients would like to receive as much information as possible. Last but not least, the efficacy of pharmaceutical care varied a lot because of patient's individual social demographic characteristics, disease, and treatment. Therefore, patient's individual difference and individual measurement should be paid attention to in the implementation in the process of comprehensive pharmaceutical care service.

It was a single-center study conducted in a tertiary hospital. The number of cases is not much bigger, which is limitation that should be considered. The results need to be further evaluated with multicenter prospective clinical randomized controlled trials in the future.

## 5. Conclusion

Pharmaceutical care service may improve cancer patients' chemotherapy knowledge, attitude, and practice, as well as quality of life.

## Figures and Tables

**Table 1:** Comparison of patients' demographic and clinical characteristics.

Characteristic	Control group (*n* = 72)	Intervention group (*n* = 77)	*X* ^2^	*P* ^a^
Sex			0.018	0.892
Male	41 (56.9)	43 (55.8)		
Female	31 (43.1)	34 (44.2)		
Age, years			1.087	0.297
≤50	35 (48.6)	44 (57.1)		
>50	37 (51.4)	33 (42.9)		
Marital status			1.577	0.209
Single	9 (12.5)	5 (6.5)		
Married	63 (87.5)	72 (93.5)		
Education level			0.671	0.715
Low	41 (56.9)	48 (62.3)		
Middle	27 (37.5)	24 (31.2)		
High	4 (5.6)	5 (6.5)		
Medical expense			2.366	0.500
Own expense	8 (11.1)	5 (6.5)		
Rural medical cooperation	19 (26.4)	24 (31.2)		
Medical insurance	38 (52.8)	44 (57.1)		
Public-funded	7 (9.7)	4 (5.2)		
Chemotherapy cycles			0.677	0.713
0	43 (59.7)	49 (63.6)		
1	16 (22.2)	13 (16.9)		
2	13 (18.1)	15 (19.5)		
Tumor staging			3.177	0.529
Unknowns	0 (0)	2 (2.6)		
I	7 (9.7)	5 (6.5)		
II	12 (6.7)	9 (11.7)		
III	18 (25.0)	20 (26.0)		
IV	35 (48.6)	41 (53.2)		

Data presented as *n* (%) patients.

^a^Between-groups comparison (control versus intervention group, before study), Chi-squared test.

**Table 2:** Patients' knowledge, attitude, and practice scores at pre- and poststudy time in two groups.

Score	Control group (*n* = 72)			Pharmaceutical intervention group (*n* = 77)			Between groups	
	Before study	After study	*P* ^*∗*^	Before study	After study	*P* ^*∗*^	*P* ^#^	*P* ^■^
Knowledge score	10.93 ± 3.83	11.86 ± 3.24	0.00	9.94 ± 3.84	17.71 ± 2.11	0.00	0.16	0.00
Attitude score	15.99 ± 3.84	16.04 ± 3.84	0.10	15.64 ± 3.92	22.04 ± 12.38	0.00	0.58	0.00
Practice score	4.89 ± 0.78	4.92 ± 0.78	0.16	4.82 ± 0.84	5.69 ± 0.47	0.00	0.60	0.00

Data presented as mean ± SD.

^*∗*^Within-group comparison (prestudy versus poststudy scores), paired Student's *t*-test.

^#^Between-groups comparison (control versus intervention group, before study), independent Student's *t*-test.

^■^Between-groups comparison (control versus intervention group, after study), Analysis of Covariance.

**Table 3:** Scores of all scales and items of the EORTC QLQ-C30 questions before and after study in two groups.

Variable	Control group (*n* = 72)			Intervention group (*n* = 77)			Between groups	
	Before study	After study	*P* ^*∗*^	Before study	After study	*P* ^*∗*^	*P* ^#^	*P* ^■^
Overall quality of life scales								
Global QOL	54.23 ± 19.20	52.78 ± 18.92	0.321	52.16 ± 18.75	59.09 ± 18.35	0.000	0.932	0.000
Function								
Physical functioning	64.25 ± 19.39	64.54 ± 18.44	0.928	63.80 ± 19.81	65.28 ± 17.52	0.597	0.889	0.789
Role functioning	49.77 ± 30.32	52.32 ± 30.04	0.625	47.40 ± 30.11	51.94 ± 29.49	0.410	0.634	0.867
Emotional functioning	59.61 ± 17.28	60.19 ± 16.68	0.340	58.1 ± 17.68	80.00 ± 17.50	0.000	0.604	0.000
Cognitive functioning	67.59 ± 17.89	67.13 ± 18.12	0.159	67.31 ± 17.61	68.83 ± 15.38	0.109	0.924	0.045
Social functioning	35.88 ± 22.14	36.57 ± 21.96	0.471	35.28 ± 21.79	36.36 ± 20.72	0.096	0.868	0.759
Symptom scales								
Fatigue	46.63 ± 23.59	45.86 ± 22.40	0.260	47.95 ± 19.69	39.73 ± 22.44	0.001	0.712	0.001
Nausea and vomiting	37.04 ± 22.59	37.73 ± 22.55	0.260	37.23 ± 22.44	30.95 ± 19.05	0.001	0.958	0.000
Pain	31.25 ± 19.37	31.22 ± 19.44	0.321	30.95 ± 18.87	23.38 ± 16.28	0.000	0.924	0.000
Single items								
Dyspnea	28.70 ± 27.01	27.31 ± 25.22	0.260	26.84 ± 23.59	26.41 ± 22.52	0.321	0.654	0.523
Sleep disturbance	54.63 ± 25.83	51.85 ± 26.76	0.109	55.41 ± 25.71	25.54 ± 23.51	0.000	0.853	0.000
Appetite loss	55.09 ± 27.50	56.48 ± 26.03	0.321	56.28 ± 26.64	53.68 ± 26.58	0.109	0.790	0.064
Constipation	34.70 ± 28.21	35.18 ± 27.91	0.784	37.66 ± 28.79	29.00 ± 27.22	0.000	0.530	0.001
Diarrhea	29.17 ± 27.94	27.78 ± 27.40	0.083	30.30 ± 28.71	19.91 ± 24.34	0.013	0.807	0.021
Financial difficulties	77.78 ± 25.63	78.24 ± 25.12	0.321	78.79 ± 25.31	79.22 ± 24.21	0.567	0.809	0.977

Data presented as mean ± SD.

^*∗*^Within-group comparison (prestudy versus poststudy scores), paired Student's *t*-test.

^#^Between-groups comparison (control versus intervention group, before study), independent Student's *t*-test.

^■^Between-groups comparison (control versus intervention group, after study), Analysis of Covariance.
